# Successful Treatment of an Atypical Mycobacterial Exit Site Infection in a Patient on Peritoneal Dialysis: A Case Report

**DOI:** 10.7759/cureus.116233

**Published:** 2026-09-14

**Authors:** Michael Shye, Anita D Sircar, Ramya Malchira, Carl Schulze, William J Martin

**Affiliations:** 1 Nephrology, University of California, Los Angeles, Los Angeles, USA; 2 Infectious Diseases, University of California, Los Angeles, Los Angeles, USA; 3 Rheumatology, University of California, Los Angeles, Los Angeles, USA

**Keywords:** atypical mycobacterium, end stage renal disease (esrd), exit site infection, peritoneal dialysis complication, peritoneal dialysis (pd)

## Abstract

Atypical mycobacterial infections are a rare cause of exit site infections in patients on peritoneal dialysis (PD) but can pose a significant risk of morbidity and frequently require catheter removal. We report a 35-year-old woman with end-stage renal disease (ESRD) due to lupus nephritis on immunosuppression and recent antibiotic use for two urinary tract infections and *Proteus mirabilis* peritonitis who developed an exit site infection due to *Mycobacterium fortuitum*,a rapidly growing mycobacterium (RGM). The patient was successfully treated with oral antibiotics without the need for PD catheter removal.

## Introduction

Peritoneal dialysis (PD) requires the placement of an abdominal catheter to allow for the clearance of toxins and removal of fluid in patients with end-stage renal disease (ESRD). After placement, the exterior portion of the catheter and exit site become colonized with bacteria and are prone to infection. Risk factors associated with the development of exit site infection (ESI) include poor exit site care, catheter displacement from exit site trauma, mechanical compression by a waist belt/clothing, swimming, and the presence of pets during treatment [1]. The risk of ESI has decreased with topical antimicrobial prophylaxis at the exit site [2].

While atypical mycobacterial ESIs remain rare, they do carry significant risk for morbidity and mortality, especially since there can be a delay in the identification of the bacteria and initiation of treatment. We report a case of a patient with ESRD on PD with risk factors for infection due to uncommon organisms who developed an atypical mycobacterial ESI and was successfully treated with antimicrobial therapy without catheter removal. This case highlights the importance of a thorough evaluation to identify when prompt catheter removal is required or when antibiotic therapy alone can be successful.

## Case presentation

A 35-year-old female with ESRD due to lupus nephritis on PD for one year was seen during a monthly PD visit complaining of persistent serosanguinous discharge for one week after minor trauma from tugging the PD catheter. Vital signs were unremarkable with temperature 98.1°F, blood pressure 138/78 mmHg, heart rate 87 beats per minute, and oxygen saturation of 98% on room air. The abdomen had normal bowel sounds and was non-tender with a PD catheter exiting the right lower quadrant. There was no erythema, rash, crusting, or tenderness at the exit site. With manual compression, a small amount of pink fluid was expressed. 

The patient was previously treated with seven-day courses of cephalexin 500mg per oral twice daily for two urinary tract infections over the past three months. The urine culture for the first episode was mishandled and the urine culture for the second infection grew *Klebsiella pneumoniae* only resistant to ampicillin. Symptoms for both urinary tract infections resolved completely with antibiotic treatment. The patient was completing a three-week course of intraperitoneal cefazolin for peritonitis due to *Proteus mirabilis* and was also taking fluconazole 100mg per oral once daily for fungal prophylaxis. The patient denied fever, abdominal pain, pain at the exit site, dysuria, or changes in urinary or bowel habits. Daily gentamicin cream was used for ESI prophylaxis. She had a remote medication allergy to ciprofloxacin causing neck pain. Home medications included mycophenolate 500mg per oral twice daily, levothyroxine 75mcg per oral once daily, metoprolol XL 25mg per oral once daily, atorvastatin 20mg per oral once daily, and cholecalciferol 2000 IU per oral once daily.

Routine bacterial Gram stain and culture of the exit site were obtained. On day one, Gram stain returned with rare gram-positive organisms with many white blood cells (WBC). On day four, the preliminary culture result showed heavy growth with acid-fast bacilli (AFB). On day seven, ultrasound of the exit site showed a 1.1 cm area of increased vascularity consistent with inflamed tissue without evidence of abscess (Figure 1). On day 25, AFB was identified as *Mycobacterium fortuitum*. Infectious disease consultation was obtained, and on day 35, antibiotic susceptibility results showed sensitivity to amikacin, ciprofloxacin, imipenem, linezolid, and moxifloxacin; intermediate susceptibility to cefoxitin; and resistance to doxycycline, trimethoprim/sulfamethoxazole, and tobramycin (Table 1). Of note, cell count, Gram stain, and culture of the peritoneal fluid did not show any evidence for peritonitis. 

**Figure 1:**
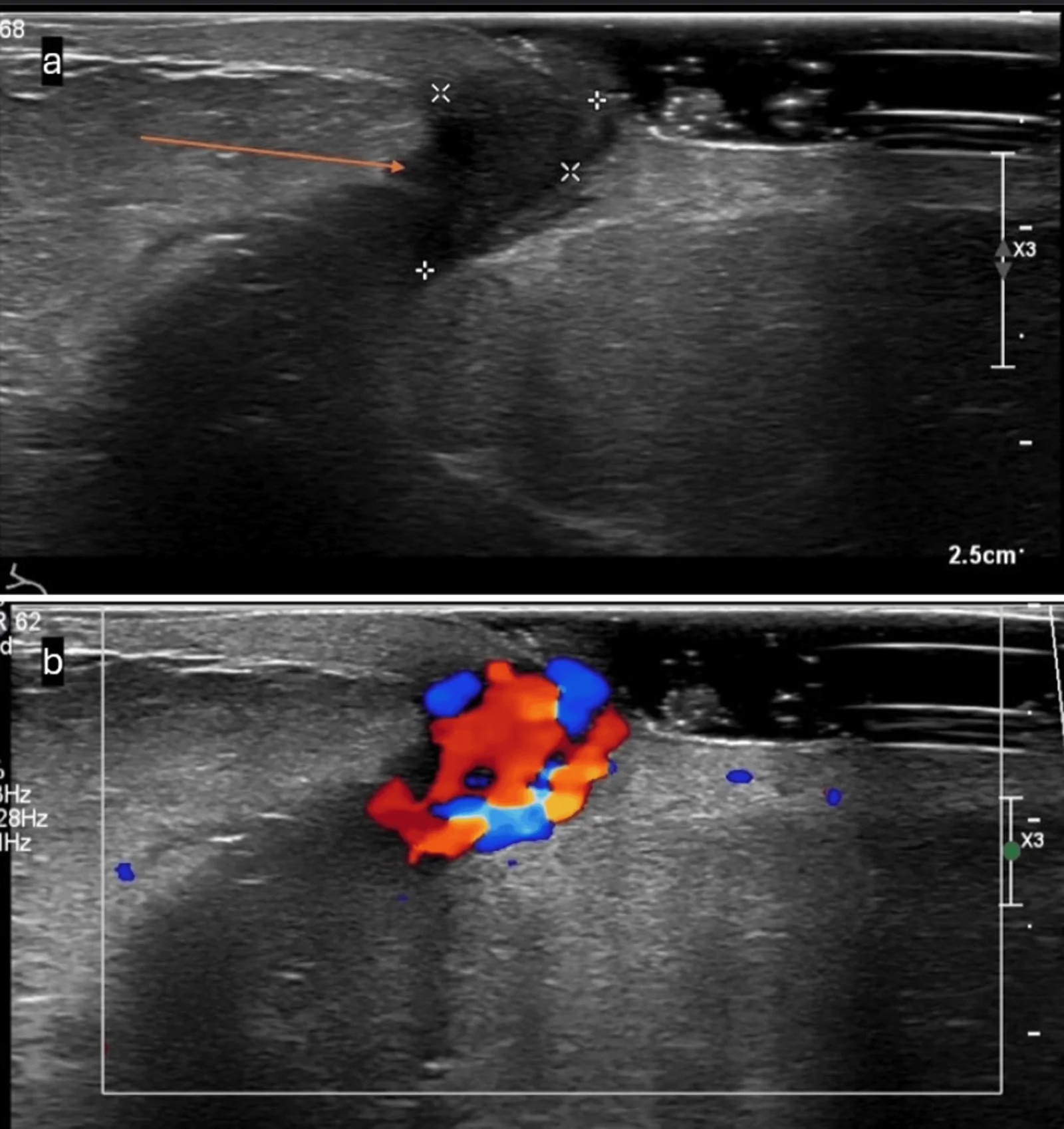
Ultrasound of exit site shows (a) 1.1 x 0.7 cm hypoechoic area just adjacent to the catheter insertion site within the subcutaneous tissues which (b) fills with color and may represent an area of inflamed subcutaneous tissue.

**Table 1:** Rapid Grower Broth Susceptibility for M. fortuitum in our patient's case.

Antibiotic	Susceptibility	Minimum inhibitory concentration
Amikacin	Susceptible	1.0 ug/mL or less,
Cefoxitin	Intermediate	32.0 ug/mL
Ciprofloxacin	Susceptible	0.12 ug/mL or less
Clarithromycin	Resistant	>16.0 ug/mL
Clofazimine		0.12 ug/mL
Doxycycline	Resistant	>8.0 ug/mL
Imipenem	Susceptible	4.0 ug/mL
Linezolid	Susceptible	2.0 ug/mL
Moxifloxacin	Susceptible	0.06 ug/mL
Tigecycline		0.06 ug/mL
Tobramycin	Resistant	8.0 ug/mL
Trimethoprim/Sulfa	Resistant	4/76 ug/mL

On day 42, exudate remained persistent at the PD exit site. Linezolid 600mg per oral twice daily and moxifloxacin 400mg per oral once daily were given for three months without recurrence of infection two years after treatment completion. The patient continued PD without interruption through the infectious course and remains active on the kidney transplant wait list. A timeline of the patient’s clinical course is summarized in Figure 2. 

**Figure 2:**
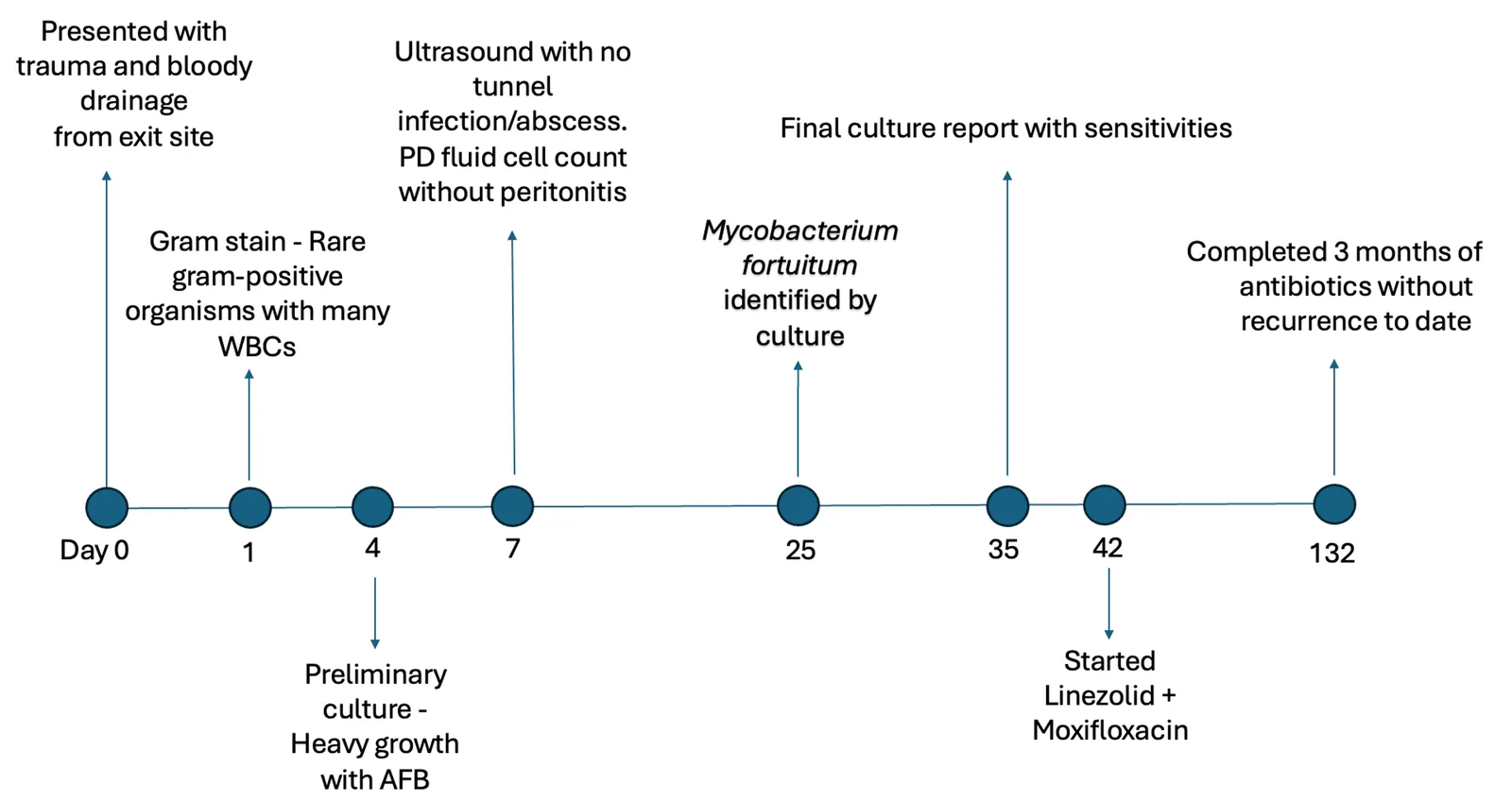
Timeline of the patient’s clinical course, including serial exit-site Gram stains and cultures from day 0 through day 132. PD: peritoneal dialysis; AFB: acid-fast bacilli

## Discussion

The diagnosis of an ESI is made clinically and is defined by the presence of purulent discharge with or without erythema of the skin at the catheter epidermal interface. Erythema, skin induration, and crusting alone do not necessarily indicate an infection. Erythema may occur immediately after catheter placement or following minor trauma such as tugging on the catheter. Some patients may also develop an allergic reaction to the cleaning agent or dressing materials. Patients with erythema, induration, and crusting should be followed closely for progression to ESI. In contrast, tunnel infection is diagnosed when tenderness, edema, erythema, or induration is present along the catheter tunnel. As with ESI, a tunnel infection is diagnosed clinically and can be made even in the absence of a fluid collection on ultrasound [3].

Exit-site discharge is sent for culture and Gram stain. Ultrasound of the catheter tract should be obtained to evaluate for peri-catheter fluid collection if a tunnel infection is suspected by physical examination with tenderness along the catheter pathway or drainage from the exit site after milking the tract. Concomitant peritonitis should be considered in patients with cloudy effluent and/or abdominal pain [4,5].

While our patient presented with exit-site discharge described as serosanguineous rather than purulent, ESI remains a clinical diagnosis. The diagnosis of ESI was made based on the following findings: Gram stain with many WBCs, preliminary culture with heavy growth of AFB, ultrasound with subcutaneous inflammation at the exit site, and persistent discharge even by day 42 when antibiotic therapy was started. 

As highlighted in one study with 44 cases of ESI with or without tunnel infection, the most common cause was gram-positive bacteria (59%, including *Staphylococcus aureus* 18%), followed by gram-negative bacteria (23%, including *Pseudomonas aeruginosa* 14%), fungus (7%), and culture negative (11%) [6]. ESI can rarely be caused by *Burkholderia cepacia*, nondiphtheria *Corynebacteria*, and atypical mycobacteria [7-9]. Over a five-year period, one Asian multi-ethnic single-center study reported that atypical or nontuberculous mycobacteria (NTM) constituted 3% of PD-associated infections [10]. 

NTM are more likely to cause infection in patients with impaired cell-mediated immunity either due to underlying diseases like HIV, or immunosuppressive therapies such as those used in cancer and solid-organ transplants [11]. One case series also suggested that NTM ESI may become more likely as a result of selection pressure for uncommon organisms with prolonged systemic antibiotic therapy in the preceding months and the use of topical gentamicin for prophylaxis or treatment [9]. Our patient presented with risk factors for NTM infection including mycophenolate use for lupus nephritis, recent antibiotic use for two urinary tract infections and peritonitis, and prophylactic topical gentamicin use.

NTM are ubiquitous environmental organisms and have been isolated from soil, water, terrestrial and aquatic animals, hospital environments, contaminated reagents and pharmaceuticals. NTM can be misidentified as diphtheroids or *Corynebacterium* species which can lead to a delayed diagnosis. When NTM is considered, Ziehl-Neelsen staining for AFB and culture on specific media should be pursued [12]. In our patient’s case, gram-positive rods were initially seen on routine Gram stain. This was followed by AFB testing that revealed heavy growth of an organism, later identified as *M. fortuitum* on ID sequencing (LabCorp, Burlington, NC, USA).

NTM causing ESI typically belong to the Runyon classification group IV, rapidly growing NTM and include *M. fortuitum*, *M. chelonae*, and *M. abscessus*. Colony growth generally occurs within seven days. A literature review found that PD-associated ESIs due to rapidly growing NTM were identified as *M. fortuitum* (58.2%), *M. chelonae* (28.2%), and *M. abscessus* (28.2%) [13].

Treatment of NTM PD-associated ESI is dependent on identification of the particular species and in vitro antimicrobial susceptibility testing. Routine broth microdilution susceptibility testing of all RGM isolates should be conducted per The Clinical and Laboratory Standards Institute (CLSI) recommendations. Testing should be done against amikacin, cefoxitin, ciprofloxacin, clarithromycin, doxycycline, imipenem, and sulfamethoxazole (or trimethoprim/sulfamethoxazole), other agents such as linezolid, moxifloxacin, and tigecycline also frequently show in vitro activity against these organisms [14]. 

*M. fortuitum* is historically considered the most susceptible to antimicrobial drugs in the RGM group, with isolates frequently being susceptible to several agents tested. Guidelines by the American Thoracic Society and the Infectious Diseases Society of America in 2007 and more recently by the International Society of Peritoneal Dialysis in 2023 recommend treatment with two antimicrobial agents with *in vitro *activity for a minimum of four months to increase the likelihood of cure [5,12]. 

Despite the recommendation of antimicrobial therapy for a minimum of four months, our patient was successfully treated with moxifloxacin and linezolid for three months only after careful consideration. After two months of treatment, the exit site was dry and healed without discharge. At that time, the decision was made to shorten treatment duration to minimize the risk of antibiotic side effect given the patient’s history of neck pain on ciprofloxacin and an increased reported rate of adverse events with combined linezolid and levofloxacin use including electrocardiogram QT prolongation, hypokalemia, peripheral neuropathy, anemia, and increased liver enzyme levels [15]. Of note, a systematic literature review from 2024 showed that the average duration of antimicrobial therapy for PD catheter-related infections without peritonitis was 3.8 months (range, 0-24 months) [16] and there is a lack of randomized controlled trials to evaluate the impact of antimicrobial treatment duration on prognosis.

Cases that fail to resolve with the recommended antibiotic course, present with abscess formation, or are caused by *M. abscessus* require surgical intervention. Simultaneous catheter removal and reinsertion can be considered but there is little data regarding clinical outcomes. ESIs due to *M. abscessus* require catheter removal due to high treatment failure rates and high morbidity and mortality rates [5,12].

In a systematic literature review of PD-related NTM infections from 2024, 130 patients were identified to have ESI with or without tunnel infection and without peritonitis. Thirty-two (24.6%) of these patients required antibiotic therapy alone without surgical intervention. The one death reported in the review was noted in the antibiotic-only group. Data regarding the particular species of NTM causing the reported cases was not provided [16].

## Conclusions

While atypical mycobacterial ESIs in PD remain rare, they carry a high risk for treatment failure, morbidity, and mortality. Risk factors for NTM ESI include an immunosuppressed state and selection pressure from recent antibiotic exposure. An ultrasound of the catheter for abscess formation and tunnel infection, evaluation of dialysis effluent for concomitant peritonitis, and identification of the mycobacterial species are crucial to assess for the risk of morbidity as well as the risk for treatment failure with oral antibiotics alone without catheter removal.
